# Rape survivors in South Africa: analysis of the baseline socio-demographic and health characteristics of a rape cohort

**DOI:** 10.1080/16549716.2020.1834769

**Published:** 2020-12-14

**Authors:** Naeemah Abrahams, Shibe Mhlongo, Esnat Chirwa, Carl Lombard, Kristin Dunkle, Soraya Seedat, Andre Pascal Kengne, Bronwyn Myers, Nasheeta Peer, Claudia M. García-Moreno, Rachel Jewkes

**Affiliations:** a Gender & Health Research Unit, South African Medical Research Council, Cape Town, South Africa; b School of Public Health and Family Medicine, Faculty of Health Sciences, University of Cape Town, Cape Town, South Africa; c Biostatistics Unit, South African Medical Research Council, Cape Town, South Africa; d Anxiety and Stress Disorder Unit, University of Stellenbosch University of Stellenbosch, Cape Town, South Africa; e Non-Communicable Disease Research Unit, South African Medical Research Council, Cape Town, South Africa; f Alcohol, Tobacco and Other Drug Research Unit, South African Medical Research, Council, Cape Town, South Africa; g Division of Addiction Psychiatry, Department of Psychiatry and Mental Health, University of Cape Town, Cape Town, South Africa; h Department of Sexual and Reproductive Health and Research, World Health Organization (WHO), Geneva, Switzerland; i School of Public Health, Faculty of Health Sciences, University of the Witwatersrand, Johannesburg, South Africa

**Keywords:** Rape, sexual violence, violence against women, gender-based violence

## Abstract

**Background:**

Little is known about women who have experienced a recent rape, and how they differ from women without this exposure. Identifying factors linked to rape is important for preventing rape and developing effective responses in countries like South Africa with high levels of sexual violence.

**Objective:**

To describe the socio-demographic and health profile of women recently exposed to rape and to compare them with a non-rape-exposed group.

**Methods:**

The Rape Impact Cohort Evaluation Study (RICE) enrolled 852 women age 16–40 years exposed to rape from post-rape care centres in Durban (South Africa) and a control group of 853 women of the same age range who have never been exposed to rape recruited from public health services. Descriptive analyses include logistic regression modelling of socio-demographic characteristics associated with recent rape exposure.

**Results:**

Women with recent rape reported poorer health and more intimate partner violence than those who were not raped. They had a lower likelihood of having completed school (Odds Ratio [OR] 0.46 95% Confidence Interval (CI): 0.24–0.87) and dependence on a government grant as a main source of income (OR 0.61: 95%CI 0.49–0.77). They were more likely to live in informal housing (OR 1.88 95%CI: 1.43–2.46) or rural areas (OR 2.24: 95%CI 1.61–3.07) than formal housing areas – however they were also more likely to report full-time employment (OR 4.24: 95%CI 2.73–6.57).

**Conclusion:**

The study shows that structural factors, such as lower levels of education, poverty, and living in areas of poor infrastructure are associated with women’s vulnerability to rape. It also shows possible protection from rape afforded by the national financial safety net. It highlights the importance of safe transportation in commuting to work. Preventing rape is critical for enabling women’s full social and economic development, and structural interventions are key for reducing women’s vulnerability.

## Background

Rape is a major human rights violation that affects many women and girls globally [1,2]. Population-based studies across six countries in Asia and the Pacific found that 4.4–41.7% of men reported ever perpetrating rape [3]. In South Africa, population-based prevalence studies have found that between 28% and 37% of men reported ever perpetrating rape, while 12–25% of women report ever experiencing rape [4,5]. Under-reporting of rape to the police is a well-established global issue [6], and the 41 583 rapes reported to South African Police Services in the year 2018–2019, therefore, reflect only a small proportion of all rapes actually occurring [7]. Indeed, a 2009 population-based study that found only 1 in 25 of the women who disclosed lifetime rape exposure had ever reported an incident to the police [4].

Following rape, many women experience long-lasting health impacts including direct and indirect psychological and physical morbidities [8]. Psychological impacts include posttraumatic stress disorder (PTSD) and other anxiety disorders, depression, and suicidality [8–10]. In South Africa, the risk of mental health problems has been found to be higher among women with histories of rape compared to women with other trauma experiences [11–13]. Much less is known about the physical health consequences of rape. However, studies of physical and sexual intimate partner violence (IPV), which overlaps with rape, affirm a range of physical health sequelae including acquisition of STIs and HIV [14]; unplanned/unwanted pregnancy and (unsafe) abortion; and maternal and child health consequences including pregnancy loss, low birth weight and prematurity [2,15–17]. Physical injuries to the body as well as the genital area can also occur during rape and can even be fatal in the case of rape-femicide [18]. Poor health-care seeking and increased risk-taking behaviours after rape have also been described, including harmful alcohol use, drug use, tobacco use, and risky sexual behaviour [2].

Research on the structural drivers of rape and sexual violence is limited. However, a recent review on drivers of physical and sexual intimate partner violence (IPV) identified three key structural factors that impact IPV both individually and synergistically: (1) lower socio-economic status indicated by poverty, including low education and food insecurity; (2) gender inequality arising from ideologies of male privilege, and (3) wide acceptability of the use of violence in social relations [19,20]. These structural drivers, in turn, impact individual-level risk factors for IPV such as adverse childhood experiences (experiences of neglect and abuse, witnessing the abuse of mother); poor mental health, including substance misuse; and poor communication in intimate relationships [19]. Evidence for the role of these drivers can be seen from emerging studies of successful violence prevention interventions that targeted these factors [21]. This body of research, however, has not separated sexual violence or rape from other forms of gender-based violence, and it is, therefore, difficult to tease out the differential vulnerability for rape.

In South Africa, there is some information from police dockets about the socio-demographic characteristics of women reporting rape to the police, but this is limited to information collected for case investigation purposes [4]. In order to learn more about women who have experienced recent rape in South Africa, we analysed the baseline enrolment data from a longitudinal study of rape survivors. These data present an opportunity to explore the socio-demographic characteristics of women recently exposed to rape and compare them with a selected group of non-exposed women. In this paper, we explore the question: how do women recently exposed to rape differ from non-exposed women from a comparable population, in respect to social and demographic characteristics, experience of intimate partner violence, and key indicators of sexual and reproductive health and health-related behaviour.

## Methods

### Setting, population and recruitment

A full account of the Rape Impact Cohort Evaluation (RICE) study methods is presented elsewhere [22]. The study was conducted within the surrounds of the city of Durban in KwaZulu-Natal (KZN) province, and the RICE clinic was located in a tertiary hospital. We recruited rape-exposed women aged 16–40 years from sexual assault service centres. Women in the control group were recruited from the waiting rooms of primary health-care clinics in the public health service in the same locality as the sexual assault service centres. Primary health-care clinics provide a wide range of services including sexual and reproductive health services such as family planning and well-baby clinics.

The South African Criminal law (Sexual Offences and Related Matters) Amendment Act 2007 define rape as unlawful and intentional oral, anal, or vaginal penetration without consent [23]. Women recruited in this study all reported vaginal penetration rape and were recruited from five rape services. Four of the recruitment sites were Thuthuzela Care Centres (TCCs) located at public health facilities, which provide one-stop services to rape survivors [24]. The fifth recruitment site was a Crisis Centre also located at a public health hospital and linked to the TCC network of services. All five recruitment sites operated 24 hours and provided medical care, legal assistance, and a suite of support and counselling services. The study recruiters worked closely with the TCC and Crisis Centre staff to identify potential participants. Inclusion criteria were being a woman aged 16–40 years. Children under 16 years were excluded for ethical reasons. The upper age limit was chosen because the HIV incidence in women is much lower over the age of 40 years. We excluded participants if they were severely mentally distressed, mentally disabled or more than 14 weeks pregnant. We also excluded women if the first baseline interview and sample collection were beyond 20 days after the index rape event. We enrolled both HIV positive and HIV negative participants. Recruiters and/or rape centre staff introduced the study and interested women were invited to attend the RICE clinic where more detailed information was given, and consent procedures occurred. None of the women attended the RICE clinic on the same day as the rape exposure. If women consented, transport arrangements were made to bring them to the clinic when they were ready within the 20-day post-rape period. The 20-day limit was to ensure we included baseline assessment of HIV status and acute stress reactions to this traumatic experience.

**Figure 1.**
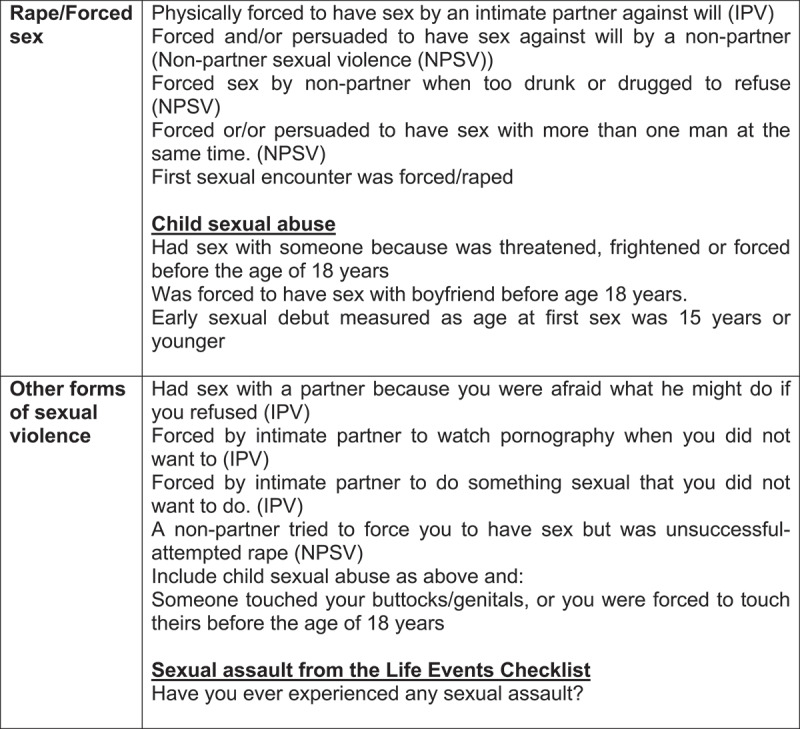
Rape/forced sex and sexual violence measured in the study

Participants in the control arm were also aged 16–40 years and the key inclusion criterion was no prior rape experience reported. We screened for rape exposure upon recruitment of the control group, and women who reported any exposure to rape or forced sex during adulthood or childhood were excluded (see Figure 1 for our definitions of rape and other forms of sexual violence measured in the study). We did not exclude those who reported attempted rape or other non-penetrative sexual violence. Recruitment started in October 2014 and ended in June 2019. This paper analyses data from 1705 participants (853 in the control group and 852 in the rape survivor group).

### Data collection and study procedures

Female field staff of similar ages interviewed participants in their preferred language at our study site using a web-based tool. Staff were trained to provide a respectful and supportive experience for participants. Female research nurses conducted clinical assessments, collected blood samples and performed on-site biological assessments for HIV and pregnancy, as well as supporting participants with a self-administered vaginal swab for testing for *Trichomonas vaginalis*. Participants received pre- and post-test counselling for each HIV test. Nursing staff also provided health promotion messages related to sexual and reproductive health. Per agreements with the National Prosecuting Authority, data on the details of the rape were not collected at the baseline interview due to concerns about data collected in the study potentially complicating legal proceedings.

#### Ethics

Ethical approval to conduct the study was obtained from the South African Medical Research Council Ethics Committee (Protocol ID: EC019-10/2013). We obtained permission to recruit women from the National and Provincial Prosecuting offices as well as the KZN Provincial Department of Health. Full consent procedures, including written informed consent, are described in the published protocol [22]. The study team included a trauma counsellor who provided immediate psychosocial support to participants on request, particularly to those who were identified as needing assistance during the study mental health assessments. Participants were then referred to the counselling services at the rape centres or could choose to return to the RICE study counsellor for additional support.

### Measures

Measures included in the study were based on the published literature and our team’s experience doing gender-based violence research in South Africa. Demographic data included age, education (classed into three categories: those who had primary education/those with secondary education and those who completed 12 years of education or more), living area (formal urban, informal urban, rural) and language spoken in the home. We assessed employment status and primary sources of income, including whether participants relied on the South African government child support grant as primary income. This grant is aimed at a primary caregiver of a child under 18 years (who reside with caregiver). A further qualification for the primary caregiver is an income below R4000.00 (US$235) per month. We used household food insecurity as a measure of poverty and asked how often people in the home go without food (coded as never/seldom vs sometimes/often). We assessed community support by asking how easy or difficult it was to borrow R200 ($12.50) in an emergency. This was coded as easy/very easy or quite difficult/very difficult.

Measures related to sexual and reproductive health included use of contraception, pregnancy history, and pregnancy complications. HIV risk factors included ever having had a sexually transmitted infection (STI) (self-reported ever had vaginal discharge/ulcers or told by nurse/doctor they had infection), early sexual debut (age at first sex was 15 years or younger), sexual partnerships (number of partners and casual partners), and transactional sexual relationships. Biological tests were done for HIV, HSV2, and *Trichomonas Vaginalis*. We followed the WHO recommendations for HIV rapid testing by conducting a confirmatory test on all positive samples, as well as an ELISA test (Vironostika HIV1/2 Elisa) [25]. HSV2 was tested using Platelia HSV (1 + 2) IgG (Biorad France) and the OSOM Trichomonas Rapid Test was used to test for *Trichomonas vaginalis* (TV) using a self-collected vaginal swab [26]. Participants who tested positive for TV were given treatment.

Current relationship status was assessed as not in a relationship, married/cohabiting, or dating not cohabiting. Sexual abuse history was assessed using a number of questions including sexual IPV, non-partner sexual violence, forced first sexual experience, lifetime trauma experience from a modified Life Events Checklist (LEC) [27] e.g. ‘have you ever experienced any sexual assault’, and experiences of child sexual violence [28] (see Figure 1). We delineated rape/forced sex and broader sexual violence experiences and ensured that women in the rape-exposed group understood that we were asking about experiences that excluded the index rape. IPV was assessed using behavioural-specific items based on studies previously done in South Africa [4] and the WHO’s multi-country study on women’s health and violence [29]. We used term ‘persuaded’ in forced sex questions as this was described by young men and women in qualitative studies on the nature of sexual violence [30]. We assessed ever and past-year exposure to IPV. Five items measured physical IPV, seven items measured emotional IPV, four items measured economic IPV, and four items measured sexual IPV. Each item was assessed as never, once, few, or many times. We also measured non-partner sexual violence as shown in Figure 1. Harmful alcohol was assessed using the Alcohol Use Disorders Identification Test (AUDIT)-C adapted for South Africa [31]. This scale had a Cronbach’s of 0.70.

### Analysis

Analyses were performed using Stata 16.0. Descriptive and bivariate analyses by exposure group were done by using percentages and means. We used Pearson’s chi-square tests and Student’s t-test to assess the difference between groups. Socio-demographic characteristics associated with rape exposure were assessed using maximum likelihood multivariable logistic regression. We considered age as a control variable and all socio-demographic variables were tested for inclusion in the model. Variables significant at p < 0.05 were retained in the model using a forward stepwise approach.

## Results

We enrolled 852 participants in the rape-exposed group and 853 in the non-exposed control group. Women were overwhelming isiZulu speaking (90%). The mean age was similar across the two groups (exposed group 25.0 years, non-exposed group 26.4 years: *p* = 0.069). Educational levels were also similar with more than a half of the participants having completed 12 or more years of schooling (exposed group 56.8% vs control group 61.1%: *p* = 0.056) (see Table 1). Although only one in five participants were employed, employment was significantly more common among rape-exposed women compared to the non-exposed control group, as was being in full-time employment (12.7% vs 3.5%: *p* = <0.001). Overall, a third of the women reported that a government child support grant was a main source of income; this was lower among the rape-exposed group than among women in the control group (27.9% vs 39.7%). A higher proportion of rape-exposed women lived in informal urban housing or in rural areas. Similar levels of food insecurity and social support (sourcing money in the community in the event of an emergency) were reported by both groups.

**Table 1.** Socio-demographic characteristics by rape exposed and non-rape-exposed group

		All participants N = 1705			Non-Rape Exposed Group n = 853			Rape Exposed Group n = 852			
Demographics	N	n	%/mean	95% CI	n	%/mean	95% CI	n	%/mean	95% CI	P value
Age (mean)	1705		25.19	24.93–25.45		25.40	25.04–25.77		24.98	24.62–25.34	0.105
Home language is isiZulu	1705	1538	90.2	88.7-.91.6	785	92.0	90.0–93.7	753	88.4	86.1–90.4	0.011
Education Primary (1–7 years) Secondary (8–11 years) Completed 12 years or more	1705	47 653 1005	2.8 38.3 58.9	2.1–3.7 36.0–40.6 56.6–61.3	17 315 521	2.0 36.9 61.1	1.2–3.2 33.8–40.2 57.8–64.3	30 338 484	3.5 39.7 56.8	2.5–5.0 36.4–43.0 53.5–60.1	0.056
Current relationship status Not currently in a relationship Currently married/Cohabiting Currently dating (not cohabiting)	1703	303 135 1265	17.8 7.9 74.3	16.1–19.7 6.7–9.3 72.2–76.3	123 68 661	14.4 8.0 77.6	12.2–17.0 6.3–10.0 74.7–80.3	180 67 604	21.2 7.9 71.0	18.5–24.0 6.2–9.9 67.8–73.9	0.001
Employed	1705	358	21.0	19.1–23.0	134	15.7	13.4–18.3	224	26.3	23.5–29.4	<0.001
Full time employed	1705	138	8.1	6.9–9.5	30	3.52	2.5–5.0	108	12.7	10.6–15.1	<0.001
Main source of income was a government child support grant	1705	577	33.8	31.6–36.1 31.6–36.1	339	39.7	36.5–43.1	238	27.9	25.02–31.1	<0.001
No income	1705	145	8.5	7.3–9.9	60	7.0	5.5–9.0	85	10.0	8.1–12.2	0.029
Settlement area	1682										<0.001
Urban–formal		1208	71.8	69.6–73.9	662	78.6	75.7–81.3	546	65.0	61.7–68.2	
Urban-Informal		280	16.7	14.9–18.5	111	13.2	11.1–15.6	169	20.1	17.5–23.0	
Rural		194	11.5	10.1–13.2	69	8.2	6.5–10.3	125	14.9	12.6–17.45	
Food security:											
Sometimes or often go without food	1704	324	19.01	17.2–21.0	155	18.17	15.7–20.9	169	19.9	17.3–22.7	0.375
Finding money for an emergency											
Difficult to find money	1705	669	39.24	36.9–41.6	343	40.21	37.0–43.5	326	38.3	35.1–41.6	0.410

Table 2 presents sexual and reproductive health measures. Some experiences were similar across the two groups: for example, a similar proportion of women in both groups reported ever having had sexual experiences (exposed group 95.6%, non-exposed group 94.8%) and ever having been pregnant (exposed group 75.1%, unexposed group 78.4%). The rape-exposed women were less likely to be using contraception (39.1% vs 32.9%: *p* = 0.007), and more likely to have had a still-birth or a miscarriage (23.0% vs 15.1%). Rape-exposed women also had greater exposure to HIV infection via past risky behaviours such as a younger age of sexual debut (*p* = <0.001), higher number of lifetime sexual partners (*p* = 0.005) and more than three sexual partners in the last year (*p* = <0.001). The rape-exposed women were also significantly more likely to test positive for HIV (48.2% vs 37.6%: *p* = <0.001) and HSV2 (73.5% vs 66.9%: *p* = 0.003) at baseline. Rape-exposed women were much more likely than those not exposed to drink alcohol (59.6% vs 45.8%: *p* = <0.001) including drinking to harmful levels (36.2% vs 26.0%: *p*-<0.001).

**Table 2.** Descriptive statistics of sexual, reproductive and health outcomes by exposure group

		All participants N = 1705			Non-Rape Exposed Group n = 853			Rape Exposed Group n = 852			
	N	n	%	95% CI	n	%	95% CI	n	%	95% CI	P value
Ever had sex	1703	1622	95.2	94.1–96.2	808	94.8	93.1–96.2	814	95.7	94.1–96.8	0.429
Currently not using contraception	1703	613	36.0	33.8–38.3	280	32.9	29.8–36.1	333	39.1	35.9–42.5	0.007
Ever pregnant	1703	1307	76.8	74.7–78.7	668	78.4	75.5–81.0	639	75.1	72.1–77.9	0.105
Ever had a still birth or a miscarriage^1^	1307	248	19.0	16.9–21.2	101	15.1	12.6–18.1	147	23.0	19.9–26.4	<0.001
Ever had a sexual transmitted Infection^2^	1703	808	47.5	45.1–49.8	378	44.4	41.1–47.7	430	50.5	47.2–53.9	0.011
Positive *Trichomonas* test^3^	1386	50	3.6	2.7–4.7	27	3.7	2.5–5.3	23	3.5	2.4–5.3	0.905
Age at 1st sex under was 15 years and younger	1705	166	9.7	8.4–11.2	52	6.1	4.7–7.9	114	13.4	11.3–15.8	<0.001
Has had 5+ partners ever	1705	490	28.7	26.6–30.9	219	25.7	22.9–28.7	271	31.8	28.8–35.0	0.005
Has had 3+ partners in the last year	1705	69	4.1	3.2–5.1	15	1.8	1.1–2.9	54	6.3	4.9–8.2	<0.001
Had a once off partner in last year	1701	452	26.6	24.5–28.7	236	27.7	24.8–30.8	216	25.4	22.6–28.5	0.279
Transactional sex with main partner	1705	168	9.85	8.5–11.4	69	8.1	6.4–10.1	99	11.6	9.6–14.0	0.014
Transactional sex with casual partner	1705	161	9.4	8.1–10.9	67	7.85	6.2–9.9	94	11.0	9.1–13.3	0.025
Transactional sex main and/or casual)	1705	237	13.9	12.3–15.6	100	11.7	9.7–14.1	137	16.1	13.8–18.7	0.009
HSV2 positive	1703	1196	70.2	68.0-.72.4	571	66.9	63.7–70.0	625	73.5	70.5–76.4	0.003
HIV positive	1705	733	43.0	40.7–45.4	322	37.8	34.6–41.1	411	48.	44.90–51.6	<0.001
Drinks alcohol	1705	899	52.7	50.4–55.1	391	45.8	42.5–49.2	508	59.6	56.3–62.9	<0.001
High AUDIT – C (1/0)	1705	530	31.1	28.9–33.3	222	26.0	23.2–29.1	308	36.2	33.0–39.4	<0.001

^1^Among ever pregnant women ^2^Women asked about ever having a vaginal discharge/ulcer, ever told by a health worker they have a STI ^3^Menstruating women not tested

Women’s experiences within their intimate relationships are presented in Table 3. Nearly two-thirds of the participants were currently in non-cohabiting relationships, and this was more common among the rape-exposed women. IPV was generally high but significantly higher among the rape-exposed women compared with the non-exposed women. Past-year IPV experience was nearly double among the rape-exposed women (emotional IPV 18.7% vs 10.5%: *p* = <0.001; economic IPV 11.0% vs 5.5%: *p* = <0.001; physical IPV 19.2% vs 10.8%: *p* = <0.001; sexual IPV 10.7% vs 1.2%: *p* = <0.001). Three out of four of the rape-exposed women (75.1%), reported one or more forms of violence against them while this was reported among one in two (54.3%) of the non-exposed women. Half of the women in the rape-exposed group had previously been raped.

**Table 3.** Descriptive statistics of intimate partner relations by exposure group

		All participants N = 1705			Non-Rape-Exposed Group N = 853				Rape Exposed Group N = 852			
	N	n	%	75% CI	n	%		75% CI	n	%	75% CI	P value
Never been in an intimate relationship	1705	53	3.1	2.4–4.1	21	2.46		1.6–3.8	32	3.8	2.7–5.3	0.124
Intimate partner violence (IPV)												
Past year Emotional IPV	1705	240	14.1	12.5–15.8	87	10.2		8.4–12.4	153	18.0	15.5–20.7	<0.001
Ever emotional IPV	1705	746	43.8	41.4–46.1	336	39.4		36.2–42.7	410	48.1	44.8–51.5	<0.001
Past year Economic IPV	1705	136	8.0	6.8–9.4	46	5.4		4.1–7.1	90	10.6	8.7–12.8	<0.001
Ever economic IPV	1705	302	17.7	16.0–19.6	124	14.5		12.3–17.1	178	20.9	18.3–23.8	<0.001
Past year physical IPV	1705	247	14.49	12.9–16.3	90	10.6		8.7–12.8	157	18.4	16.0–21.2	<0.001
Ever physical IPV	1705	783	45.9	43.6–48.3	354	41.5		38.3–44.8	429	50.4	47.0–53.7	<0.001
Past year sexual IPV	1705	97	5.7	4.7–6.9	10	1.2		0.6–2.2	87	10.2	8.4–12.4	<0.001
Ever sexual IPV	1705	206	12.1	10.6–13.7	34	4.0		2.9–5.5	172	20.2	17.6–23.0	<0.001
Ever physical, sexual, emotional, economic IPV and non-partner sexual violence	1705	1104	64.8	62.5–67.0	463	54.3		50.9–57.6	641	75.2	72.2–78.0	<0.001
Previous rape Incident was first rape experience Previously raped	852						0	0	438 414	51.4 48.6	48.1–54.8 45.2–52.0	NA

The logistic regression model (Table 4) shows that women reporting recent rape were half as likely as those who had not to have completed school or further education (Odds Ratio [OR] 0.45: 95%CI 0.24–0.86), and they were also less likely to receive a government child support grant (OR 0.61: 95%CI 0.49–0.77). They were twice as likely to live in an area of informal housing (OR 1.88: 95%CI 1.43–2.46) or a rural area (OR 2.22: 95%CI 1.61–3.07), rather than in an urban area of formal housing. There was a more than 4-fold increased likelihood of full-time employment among the rape survivors (OR 4.24: 95%CI 2.74–6.57).

**Table 4.** Mutually adjusted logistic regression model for socio-demographic factors associated with rape exposure

	Adjusted Odds Ratio	Lower Confidence Interval	Upper Confidence Level	P-value
Age, per year	0.98	0.96	1.00	0.50
Education				
Primary completed (1–7 years)	Ref			
Secondary incomplete (8–11 years)	0.62	0.33	1.17	0.143
Completed 12 years or more	0.46	0.24	0.87	0.016
Settlement area				
Urban-formal	Ref			
Urban-informal	1.88	1.43	2.46	<0.001
Rural	2.22	1.61	3.07	<0.001
Full time employment				
No	Ref			
Yes	4.24	2.74	6.57	<0.001
Main source of income is from the government child support grant				
No	Ref			
Yes	0.61	0.49	0.77	<0.001

## Discussion

This study found that women who have experienced recent rape were more likely to be poorly educated and live in informal housing or rural areas. However, women reporting recent rape were also more likely to report full-time employment; this may be linked to their area of residence and is discussed in detail below. We also found that women reporting recent rape were less likely to report depending on the social protection safety net in the form of the government’s child support grant. After adjusting for other factors (age, settlement area, child support grant as main source of income and full-time employment), having completed secondary school or having a higher education was less likely for women recently exposed to rape. This finding is similar to that of other studies of risk factors for sexual violence and IPV that have shown reduced risk among women with secondary or higher education level [20,32]. It is likely that the mechanism of protection is related to greater income, access to social support, and differences in life opportunities for women who have school and post-school certificates [33].

Our study also showed that women reporting recent rape exposure were more likely to live in informal housing areas and rural areas, and thus confirms the widely reported association between gender-based violence, particularly IPV, and poverty [19,34–36]. In South Africa, two recent studies of IPV among young men and women in informal settlements (one conducted near our study area and one in Gauteng Province) found that both IPV and non-partner rape were much more prevalent in these environments than in general population studies [35,36]. Informal settlements are characterized by underdeveloped infrastructure, and often lack built roads, plumbing, sewage, and safe or legal electrical grids. They originally emerged in South Africa on the periphery of cities as part the apartheid system of residential segregation, control and exclusion [37], and continue to arise due to lack of affordable built housing near urban centres. The congested socio-physical environment of informal settlements with multiple deprivations creates numerous points of vulnerability to gender-based violence such as poor lighting, shared communal sanitation, and poor access to safe public transport systems. Similarly, under-development and poor infrastructure are features of the South African rural landscape [38]. Both settings have high levels of unemployment among young men, and social norms supporting gender inequality and use of violence. These provide two known key drivers of gender-based violence [35,36,39]. Our findings affirm the importance of structural interventions to prevent rape of women living in vulnerable populations in rural areas and informal settlements, as well as interventions to address the specific drivers of perpetration by men. Our findings also point to the need to provide high-quality, accessible, post-rape care services in informal settlements and rural areas. It is imperative that post-rape services reach these communities and they must be included in the country’s recently developed National Strategic Plan to Prevent Gender-Based Violence and Femicide [40].

Our study also found women reporting recent rape were more likely to have full-time employment. This may be an indicator of women’s risk of rape when travelling to and from work. The national rape justice study found 37.9% of adult rapes of women occur while using public transport or when walking in public; this points to the possibility of increased exposure for women who work [41]. It is also possible that women who work full time have more earnings and more possessions at home which makes them more vulnerable to housebreaking and rape in this context. The Rape Justice study reported that 16.8% of adult rapes occur in the context of house-breaking crimes. However, it is also possible that the control group recruited for this study may have over-represented unemployed women, as women with full-time work were not easily available for recruitment at the primary care clinics. We note that women who were employed struggled to return for follow-up interviews later in the study, as even short periods of employment were highly sought after to ensure livelihoods.

We also found that women reporting a recent rape were less likely to report the government child support grant as a main source of income. A myriad of research globally and within South Africa has shown support grants protect women and children against a range of vulnerabilities [42,43]. A recent review has shown evidence of decreased intimate partner violence associated with cash transfers [44]. Although South Africa’s child support grants are not large (about R15 ($1) per day), they do provide some economic relief and in IPV survivors it has been shown to reduce conflict and women’s dependence on male partners [43]. We did not ascertain which study participants were eligible for the grant in terms of income (less than R4000 ($235) per month), so it is possible that some of those who did not receive a grant would have been eligible. It is recognised that many of the most vulnerable women in communities do not receive grants as they may struggle to compile the documentation needed to apply for grants, including losing papers in the frequent fires that occur in informal settlements or being undocumented migrants [45].

We have shown that at the time of the rape, women had much poorer health than those who were not raped. They were less likely to be on contraception, more likely to have had a pregnancy-related problem, and more likely to ever have had a sexually transmitted infection, including HIV. They also reported more sexual partners, transactional sex, and harmful alcohol use. These health risks may stem from the greater exposure to poverty among women who experienced recent rape, as well as the fact that they were much more likely to have experienced previous rape, trauma in childhood and other trauma, and IPV. Poverty and past trauma have been shown to elevate the risk of health problems [2,46]. The finding that half of the women in the rape-exposed group reported this was not their first rape highlights the risk of revictimization, and the strong association between recent rape and past experience of IPV suggests that revictimization risk extends across multiple forms of GBV. Both findings underscore the importance of comprehensive psychosocial support for survivors of rape and IPV.

The poor sexual and reproductive health status of the women recently exposed to rape has implications for service provision as it suggests that post-rape care needs to be tailored to a population with a high prevalence of pre-existing vulnerabilities. These include substance use which is often a mechanism for coping with a history of complex trauma. Post-rape HIV services need to prevent rape-related HIV acquisition through provision of post-exposure prophylaxis, identify women who are not aware of their positive HIV status and refer them for treatment, and ensure that HIV-positive women already on ART have support to continue their medication in the aftermath of the rape. The pregnancy risk from rape is elevated by the low rates of contraception use and underscores the need for post-rape services to offer emergency contraception.

Our study has limitations. We recruited a volunteer sample which is not necessarily representative of the total population of women seeking post-rape care. Further we did not include male victims, girls under 16 years or women over the age of 40 years. The comparison population was intended to be as similar as possible to the rape-exposed population. Because the rape-exposed women were actively seeking services, we used health service users as comparison group with the intention that comparing two groups of services would eliminate some of the biases that could otherwise arise, however, this limit generalizability to non-service users. We initially planned to age match the control recruits, and also attempted to recruit controls from primary health services in the same location as the address of the recruited exposed women, but the rate of recruitment and logistical constraints impacted on the study budget which required us to adapt our initial strategy. However, the mean age and range was very similar in the two study arms (mean age 24.97 vs 25.36). The challenges related to disclosure of rape were shown in this study when we found 94 women recruited into the control group had experienced rape in the past (excluded from this analysis) despite our screening processes. Discussion with the research team suggests that this resulted from combination of our screening processes (e.g. recruitment at clinic waiting rooms prevented one-on-one question and answer sessions), participants’ understanding of sexual violence/rape and women’s’ deliberately withholding information to become part of our study. We plan to explore this further with qualitative methods. Despite these limitations, this study is the first in a developing country setting to report on a large sample of recently rape exposed women and a control group. While socio-demographic data are sometimes collected at post-rape care services for administrative purposes, this information is seldom analysed to assist with understanding risk factors or informing prevention.

## Conclusion

This is the first study that enrolled a large sample of women seeking health services for a recent rape in a developing setting and compared baseline socio-demographic and health characteristics with a comparative sample of women seeking general healthcare services. The study baseline data highlights the complexity of the relationship between poverty (informal housing, rural areas), education, income support (in the form of child support grants) and rape. Contrary to what would be expected, employment did not confer protection, which may be related to the nature of the employment and lack of access to safe transportation. Our findings also highlight the high prevalence of revictimization among women reporting recent rape, with a high prevalence of previous experience of rape and intimate partner violence. Overall, the study confirms that prevention of rape is critical for women’s health and development and that structural interventions must form part of the response to rape. This is essential for achieving the Sustainable Development Goals (SDGs), particularly SDG 5 on gender equality and empowerment of women.

## Paper context

Not much is known about rape survivors in developing settings. The Rape Impact Cohort Evaluation study (RICE) compared the socio-demographic and health profile of recently rape-exposed women with a comparative sample of non-exposed women. The study shows the complex relationship between poverty, education, income support, and rape. The study confirms prevention of rape is critical for women’s health and development and that structural interventions must form part of the response to rape.
